# The influence of information processing speed on memory processes in patients with relapsing-remitting and primary progressive multiple sclerosis

**DOI:** 10.1038/s41598-025-96181-6

**Published:** 2025-04-08

**Authors:** Carolin Balloff, Sven G. Meuth, Heinz Wiendl, Andreas Johnen, Jens Bölte, Iris-Katharina Penner, Nils C. Landmeyer

**Affiliations:** 1 Department of Neurology, Medical Faculty and University Hospital Düsseldorf, Heinrich Heine University, Moorenstrasse 5, 40225 Düsseldorf, Germany; 2 Department of Neurology, University Hospital Münster, University of Münster, Albert-Schweitzer-Campus 1, 48149 Münster, Germany; 3 Department of Neurology, Kliniken Maria Hilf GmbH, Mönchengladbach, Germany; 4 Cogito Center for Applied Neurocognition and Neuropsychological Research, Düsseldorf, Germany; 5 Department of Neurology and Neurophysiology, Medical Center, University of Freiburg, Freiburg, Germany; 6 Department of Psychology, University of Münster, Münster, Germany; 7 Department of Neurology, Inselspital, Bern University Hospital, University of Bern, Bern, Switzerland

**Keywords:** Multiple sclerosis, Psychology, Human behaviour, Cognitive neuroscience

## Abstract

Slowed information processing speed (IPS) is the primary cognitive impairment in people with multiple sclerosis (pwMS). Although it is known to affect memory processes, the relationships between verbal learning (VL), verbal memory (VM) and IPS in different phenotypes of multiple sclerosis (MS) remain underexplored. This bicentric study aimed to investigate in detail IPS, VL, and VM as well as the relationships between these cognitive domains in a large sample of 92 pwMS (61 with early relapsing-remitting MS (pwRRMS), 31 with primary-progressive MS (pwPPMS)) and 61 age-matched healthy controls (HC). Compared with HC, pwPPMS performed significantly worse on all cognitive measures, whereas pwRRMS performed worse than HC only on IPS and VL. Irrespective of phenotype, IPS impairment emerged as the core cognitive deficit in MS, affecting 25% of the RRMS group and 45% of the PPMS group. PwRRMS outperformed pwPPMS in all domains, with the most pronounced differences observed in VL. Isolated VL or VM impairments were rare, as most pwMS with these deficits also had concurrent IPS impairment. PwPPMS had significantly higher rates of IPS and VL impairment than pwRRMS, but not in VM. Regression analyses showed that IPS significantly predicted both VL and VM performance in both MS groups. In HC, IPS was only associated with VL performance, hinting at different cognitive mechanisms underlying the performance in this domain. This study provides evidence that pwPPMS experience more pronounced cognitive changes in IPS and VL compared to pwRRMS. It also highlights the pervasive impact of IPS on VL and VM performance in both subtypes, underscoring the importance of IPS-focused interventions in the management of MS-related cognitive dysfunction.

**Supplementary Information:**

The online version contains supplementary material available at 10.1038/s41598-025-96181-6.

## Introduction

Cognitive impairment (CI) is a debilitating condition, present in approximately 40–65% of people with multiple sclerosis (pwMS)^1,2^. Deficits typically span multiple cognitive domains, with learning, memory, and information processing speed (IPS) being consistently impaired across disease stages and phenotypes^3,4^. Slowed IPS is considered the primary and most common cognitive symptom in pwMS. Its prevalence is significantly higher than impairments in other cognitive domains and is particularly pronounced in progressive phenotypes compared with relapsing-remitting multiple sclerosis (RRMS)^3,5–8^.

Established cognitive models such as the *Cognitive Developmental Cascade* Theory^9^ describe IPS as a fundamental cognitive function which determines the rate of information uptake and thus influences all subsequent cognitive operations^9–11^. According to this theory, IPS represents the first stage of a cognitive hierarchy and directly influences working memory, which is the basis for higher cognitive abilities, such as learning and retention. Developed further and applied to pwMS, this concept led to the *Relative Consequence Model*, which suggests that reduced IPS is the fundamental deficit in pwMS, compromising working memory and other cognitive functions^5^.

Several studies have confirmed this integral role of IPS in pwMS, reporting significant influence of IPS on new learning abilities^12^ and working memory^6,13^. Performance improvements in planning and working memory have been observed when additional time is provided to process information^14,15^, further highlighting the essential role of IPS in cognitive functioning in general.

Given this critical role of IPS, it is clinically challenging to assess learning and memory processes in pwMS without the confounding effects of IPS. However, an accurate description of individual cognitive profiles is essential for tailored cognitive training programs and compensation strategies. For example, memory dysfunction requires different neuropsychological training than isolated IPS dysfunction, making it crucial to delineate these cognitive functions in pwMS.

Verbal memory (VM) tests in clinical practice typically involve recall of sequentially presented word lists, with immediate recall serving as a proxy for verbal learning (VL) efficiency. Free recall after a delay of 20–30 min can be used as a marker of efficiency in consolidating and accessing previously learned information. However, the Brief International Cognitive Assessment for Multiple Sclerosis (BICAMS)^16^, a widely used screening tool, defines verbal memory solely by the learning trials of the second edition of the California Verbal Learning Test II (CVLT-II)^17^. A delayed recall trial was not included in BICAMS because the goal was to recommend a brief instrument with a maximum administration time of 15 min^16^. Previous research has shown that pwMS performed significantly worse on the learning trials compared with HC^18^, indicating that the total learning score is sensitive to MS impairment^16^. However, it is worth noting that short and long delayed recall measures may be even more sensitive, as they have demonstrated larger effect sizes when comparing pwMS and HC^18^.

The influence of IPS on VL and VM in pwMS remains largely unexplored despite its high relevance for cognitive rehabilitation. A recent study by Chiaravalloti et al.^19^ using BICAMS, found an association between IPS (via the Symbol Digit Modalities Test [SDMT])^20^ and learning scores on the CVLT-II in people with progressive MS (primary or secondary). However, the relationship between IPS and measures of delayed recall has not been investigated, and results are limited to the progressive forms of the disease. Consequently, the potential differential influence of IPS on VL and VM, as well as any variations across different MS phenotypes remain uncertain.

Therefore, the aim of the present bicentric study was to evaluate the extent and nature of verbal VL and VM deficits in relation to IPS in a large cohort of clinically well-characterized pwRRMS, pwPPMS, and closely matched healthy controls (HC). We hypothesized that pwRRMS and pwPPMS would show significant differences from HC in IPS, VM and VL performance. Furthermore, in line with the *Cognitive Developmental Cascade Theory*^9^ and the *Relative Consequence Model*^5^, we expected differential influences of IPS on VL and VM in both patient groups and HC as a potential explanation for differential impairment in these measures. We also aimed to explore potential interactions with disease subtypes, given the differences in pathophysiology between RRMS and progressive subtypes, such as the degree of neuroinflammation and deep gray-matter neurodegeneration^21,22^. We expected that pwPPMS would show greater impairments in IPS, VM and VL, with the influence of IPS on VM and VL differing between disease courses.

## Methods

This bicentric study was a retrospective evaluation of patient data collected between October 2016 and July 2019. Data were collected at the University Hospital Münster, Germany, and at the COGITO Center for Applied Neurocognition and Neuropsychological Research in Düsseldorf, Germany, by personnel trained in the administration and scoring of neuropsychological measures and questionnaires. Participants provided written informed consent prior to study participation and the study was approved by the ethics committee of the University Hospital Münster (reference number: 2017-754-f-S). The study adhered to the tenets of the Declaration of Helsinki. All data were collected in a single session starting with a brief structured interview, followed by neuropsychological tests and self-report questionnaires. Neuropsychological testing was conducted in a quiet room without disturbances, and the tests were administered in the same order for all participants according to the test manuals. Impairment for all three measures of interest (IPS, VL & VM) was defined as a z-score below z = -1.645. This equals the 5th percentile of normally distributed performance and is consistent with common research practice^23^.

### Participants

Data from 92 people diagnosed with definite MS (31 PPMS, 61 RRMS) and 61 test-naïve HC were included in the study. Exclusion criteria were as follows: (1) history of neurological diseases other than MS, (2) psychiatric disorders other than mild depression, (3) drug or alcohol abuse. Following the recommendation of Costa et al.^24^, pwMS were required to be relapse-free for at least 30 days. The control groups were matched to the RRMS and PPMS groups based on sex, age, and education.

### Information processing speed

The Rao-adapted version of the SDMT^20,25^ was used as the measure of IPS. This test has excellent psychometric properties^26^ and is included in all major test batteries of cognitive functioning in pwMS. Participants are presented with a reference table at the top of the test form, consisting of nine different symbols paired with numbers (one through nine). Below this reference table are eight rows with a total of 110 symbols with missing digit assignments. Participants are required to verbally identify the correct number for each symbol, starting at the top left and moving down as quickly as possible. The key remains visible throughout the test. After a short practice period of ten symbols, participants are instructed to work as quickly and as concentrated as possible, while the examiner records all correctly assigned digits within 90 s. Raw total scores were transformed into z-scores, correcting for age, sex and education, based on the German norms published by Scherer et al.^27^.

### Verbal learning and memory

In accordance with the German BICAMS validation^28^, VL and VM were measured using the Rey Auditory Verbal Learning Test (RAVLT, German version: Verbaler Lern- und Merkfähigkeits-Test [VLMT])^29^. In this test, the examiner reads aloud a list of 15 words which the participant must recall immediately. This procedure is repeated five times, with the number of correctly recalled items recorded for each learning trial.

After the fifth learning trial, the examiner reads aloud a second word list of 15 words (interference trial). Again, the participant is required to memorize and immediately recall as many items as possible from this new list. Immediately following this interference trial, the participant is asked to recall the original word list for a sixth time (trial 6). After a delay of 30 min, the participant is requested to recall the word list one last time (trial 7). Because the difference in the number of words recalled between trial 5 and trial 7 (Δ trial 5–7) has been suggested as the most reliable differential diagnostic measure, we used this retention score as an indicator of VM^29^. VL was operationalized as the total number of words recalled across trials 1 to 5 (ΣT1-5). Raw test scores were transformed into z-scores based on the norms provided in the manual.

### Fatigue

The Fatigue Scale for Motor and Cognitive Functions (FSMC)^30^ was used to assess fatigue. The FSMC is a 20-item self-report questionnaire with ten items addressing MS-related cognitive and motor fatigue, respectively. The items are rated on a five-point Likert scale with higher scores indicating greater perceived fatigue. The *Würzburger Erschöpfungsinventar bei Multipler Sklerose* (WEIMuS)^31^ is another self-report fatigue questionnaire for pwMS with 17 items relating to physical and cognitive symptoms. Again, items are scored on a five-point Likert scale with higher scores indicating perceived fatigue. Of the participants with missing FSMC questionnaires, 15 (13 pwPPMS, 2 pwRRMS) completed the WEIMuS. To avoid missing values in our analyses, a general fatigue scale was developed by combining the results on the two instruments into a comparable classification system. Because the WEIMuS classification system is limited to two dimensions (normal, clinically meaningful), transformation of the four-dimensional FSMC classification (no, mild, moderate, or severe fatigue) was necessary to achieve an equivalent categorization. FSMC scores classified as no or mild fatigue were summarized as normal, while moderate and severe scores were classified as clinically meaningful. Based on this transformation, a two-step fatigue score was assigned to each participant.

### Depression

The German version of the Hospital Anxiety and Depression Scale (HADS)^32^ is a 14-item self-report questionnaire with seven four-point scaled items each for anxiety and depression. Higher total scores in each domain indicate greater symptom manifestation, which is classified as normal, marginal and clinically meaningful^32^. The Beck Depression Inventory II (BDI-II)^33^ is another 21-item screening instrument for depressive symptoms and their severity. Items are scored on a four-point intensity scale with higher scores indicating more severe depression. As the HADS has been validated in MS and achieves higher sensitivity and specificity in this population^34,35^, we primarily used the HADS depression score to control for depression. HADS depression scores were available for 60 HC and 68 pwMS (57 pwRRMS, 11 pwPPMS). 17 pwMS and one HC with missing HADS data completed the BDI-II.

Parallel to the procedure used to create one comparable fatigue scale, the results of the two depression scales were transformed into a general depression classification. Because the HADS provided a three-dimensional division (normal, marginal, clinically meaningful), we transformed the five-dimensional BDI-II classification (none, minimal, mild, moderate, severe) to match the HADS categories. Scores classified as no depression on the BDI-II were equated with the first HADS category (normal), while minimal and mild BDI-II-scores were combined into the marginal category. Moderate and severe BDI-II scores were classified as clinically meaningful. Based on this transformation, a new classification of depressive symptoms was assigned to each participant.

### Statistical analysis

Statistical analyses were performed using IBM SPSS Statistics (version 29). A significance level of 0.05 (two-sided) was used for all tests. Demographic and clinical characteristics were compared between each group of pwMS and their respective matched HC group. PwPPMS and pwRRMS were also compared with each other. The Mann-Whitney U test was used for continuous variables due to non-parametric distribution in at least one subgroup. Categorical data were analysed using chi-square-test or Fisher’s exact test, depending on the expected values per cell.

Group differences in neuropsychological tests were examined using independent t-tests or Mann-Whitney U tests, chosen according to the distribution of the variables. For impairment rates in the three domains of interest (IPS, VL, VM), chi-square-tests or Fisher’s exact tests were conducted, depending on expected cell sizes. Due to the directional hypothesis of more frequent impairment in the PPMS group compared to RRMS group, one-sided p-values are reported for the neuropsychological comparisons between these two groups.

Multiple linear regression models were calculated to assess the influence of IPS and other variables on VL and VM performance. First, a set of predictors was selected based on theoretical assumptions to control factors that could influence the relationship between IPS and both VM and VL. These included age, years of formal education^1^, sex^36^, depression^37^ and fatigue^38^. Although our regression models operated with age-corrected z-scores, we decided to include age as a possible predictor because it is the most reported predictor of memory performance^39^.

Second, backward selection models were run for the different groups (RRMS, PPMS, HC) including the full set of potentially relevant predictors (see above). Significant predictors from any of the initial regression models (Tables S1-S4) were used to predict VL and VM in all groups using the enter method. Accordingly, six separate regression models were of interest: (1) predicted VL performance for RRMS, (2) predicted VM performance for RRMS, (3) predicted VL performance for PPMS, (4) predicted VM performance for PPMS, (5) predicted VL performance for HC, (6) predicted VM performance for HC. For each subgroup, the regression weights for VL and VM were compared (see Supplementary Methods for the according formulas).

## Results

### Demographic and clinical characteristics

Descriptive statistics of the sample are presented in Table 1. Indicative of successful matching, Mann-Whitney U and chi-square-tests showed no significant differences in age and sex between pwPPMS and their matched HC group. However, the PPMS group had significantly fewer years of education than the HC group. Similarly, there were no significant differences in age or between the RRMS group and their HC counterparts. Both groups of pwMS reported higher levels of depression and fatigue than their HC counterparts.

Significant differences between pwPPMS and pwRRMS were found for age, education, EDSS and fatigue. PwPPMS were older, had fewer years of formal education, and higher EDSS scores. However, the RRMS group exhibited a higher percentage of clinically meaningful fatigue.

**Table 1:** Demographics and clinical characteristics of the sample.

Characteristic	RRMS analyses			PPMS analyses			RRMS vs. PPMS
	RRMS (*n* = 61)	HC (*n* = 61)	*p*-value	PPMS (*n* = 31)	HC (*n* = 31)	*p*-value	*p*-value
Age, median (IQR), years	38 (14)	28 (28)	0.090	53 (19)	52 (9)	0.100	**< 0.001**
Sex, n (%), female	39 (63.9)	39 (63.9)	1.00	15 (48.4)	15 (48.4)	1.00	0.15
Disease duration, median (range), months	57 (101)	NA	NA	72 (121)	NA	NA	0.21
EDSS, median (IQR)	2 (2)	NA	NA	5 (2.6)	NA	NA	**< 0.001**
DMT exposure, n (%)	54 (88.5)	NA	NA	17 (54.8)	NA	NA	**< 0.001**
Education, n (%)^a^			0.16			**0.019**	0.07
≤ 9 years	0 (0)	0 (0)		2 (6.7)	0 (0)		
10–12 years	7 (11.5)	2 (3.3)		6 (20.0)	1 (3.2)		
> 12 years	54 (88.5)	59 (96.7)		22 (73.3)	30 (96.8)		
Depression, n (%)^b^			**< 0.001**			**< 0.001**	0.07
Normal	39 (66.1)	59 (96.7)		13 (50)	30 (96.8)		
Marginal	13 (21.3)	2 (3.28)		12 (46.2)	1 (3.23)		
Clinically meaningful	7 (11.5)	0 (0)		1 (3.80)	0 (0)		
Fatigue, n (%), clinically meaningful^c^	43 (72.9)	0 (0)	**< 0.001**	12 (50)	0 (0)	**< 0.001**	**0.046**

*p*-values <0 0.05 are in boldface and based on two-tailed analysis. RRMS = Relapsing Remitting Multiple Sclerosis. PPMS = Primary Progressive Multiple Sclerosis. EDSS = Expanded Disability Status Scale. DMT = disease modifying therapy. ^a^Missing data: *n* = 1 PPMS (information on secondary school diploma available (10 years), but information on subsequent years of education is missing). ^b^Missing data: *n* = 2 RRMS, *n* = 5 PPMS. Classification as follows clinically meaningful based on scores ≥ 11 for Hospital Anxiety and Depression Scale (HADS) (*n* = 60 HC, *n* = 57 RRMS, *n* = 11 PPMS) and ≥ 20 for Beck Depression Inventory (BDI-II) (*n* = 1 HC, *n* = 15 PPMS, *n* = 2 RRMS). ^c^Classification as clinical based on scores ≥ 53 in the Fatigue Scale of Motor and Cognition (FSMC)^30^ (*n* = 57 RRMS, *n* = 11 PPMS) or ≥ 32 in the Würzburger Erschöpfungsinventar bei Multipler Sklerose (WEIMUS)^31^ (*n* = 13 PPMS, *n* = 2 RRMS).

### Performance in neuropsychological measures

Neuropsychological characteristics are presented in Table 2. Mean performance did not fall below the CI cut-off of z < -1.645 on any cognitive test and in any group. However, Mann-Whitney U tests showed that matched HC significantly outperformed both pwPPMS and pwRRMS on the SDMT with medium effect sizes for both groups. Both groups of pwMS also performed significantly worse than their matched HC on VL, with small (RRMS) and medium (PPMS) effect sizes. In VM, there was no significant difference between pwRRMS and HC, but pwPPMS performed significantly worse than their matched HC with medium effect size. Comparing the two MS groups, pwRRMS performed significantly better than the PPMS group on all cognitive measures (SDMT, VL, VM), with small (VM) to medium (SDMT, VL) effect sizes.

**Table 2:** Neuropsychological test results.

	RRMS analyses				PPMS analyses				RRMS vs. PPMS	
Characteristic	RRMS (*n* = 61)	HC (*n* = 61)	*p*-value	ES	PPMS (*n* = 31)	HC (*n* = 31)	*p*-value	ES	*p-*value	ES
SDMT										
z-score, mean (SD)	-1.05 (1.06)	0.14 (1.00)			-1.57 (1.28)	0.14 (0.99)			**0.02** ^b^	0.46^c^
z-score, median (IQR)	-1.04 (1.22)	0.08 (1.53)	**< 0.001**	0.51^a^	-1.32 (1.55)	0.08 (1.67)	**< 0.001**	0.64		
Verbal learning^c^										
z-score, mean (SD)	0.49 (1.39)	1.03 (0.80)			-0.18 (1.35)	1.00 (0.81)			**0.** **02** ^b^	0.49^c^
z-score, median (IQR)	0.64 (1.96)	1.06 (1.04)	**0.027**	0.20^a^	0.03 (2.17)	1.04 (1.01)	**<0.001**	0.43		
Verbal memory^d^										
z-score, mean (SD)	-0.23 (1.30)	0.14 (0.76)			-0.71 (1.37)	0.02 (0.84)				
z-score, median (IQR)	0.19 (1.70)	0.19 (1.02)	0.43	0.07^a^	-0.40 (1.77)	0.19 (1.02)	0.**010**	0.33	**0.** **02** ^b^	0.21^a^

*p-*values < 0 0.05 are in boldface and based on two-tailed analysis if not indicated otherwise. RRMS = Relapsing Remitting Multiple Sclerosis. *PPMS* primary progressive multiple sclerosis, *HC* healthy controls, *SDMT* symbol digit modalities test, *VLMT* Verbaler Lern- und Merkfähigkeits-Test, *ES* effect size. ^a^Pearson’s r calculated based on Mann-Whitney U test statistics. ^**b**^One-sided *p-*value due to directed hypothesis. ^c^Cohen’s d. ^d^Verbal learning was defined as the total score of trial 1 to trial 5 of the VLMT. ^e^Verbal memory was defined as the difference in the number of words remembered between trial 5 and trial 7 of the VLMT.

### Impairment in neuropsychological measures

**Fig. 1:**
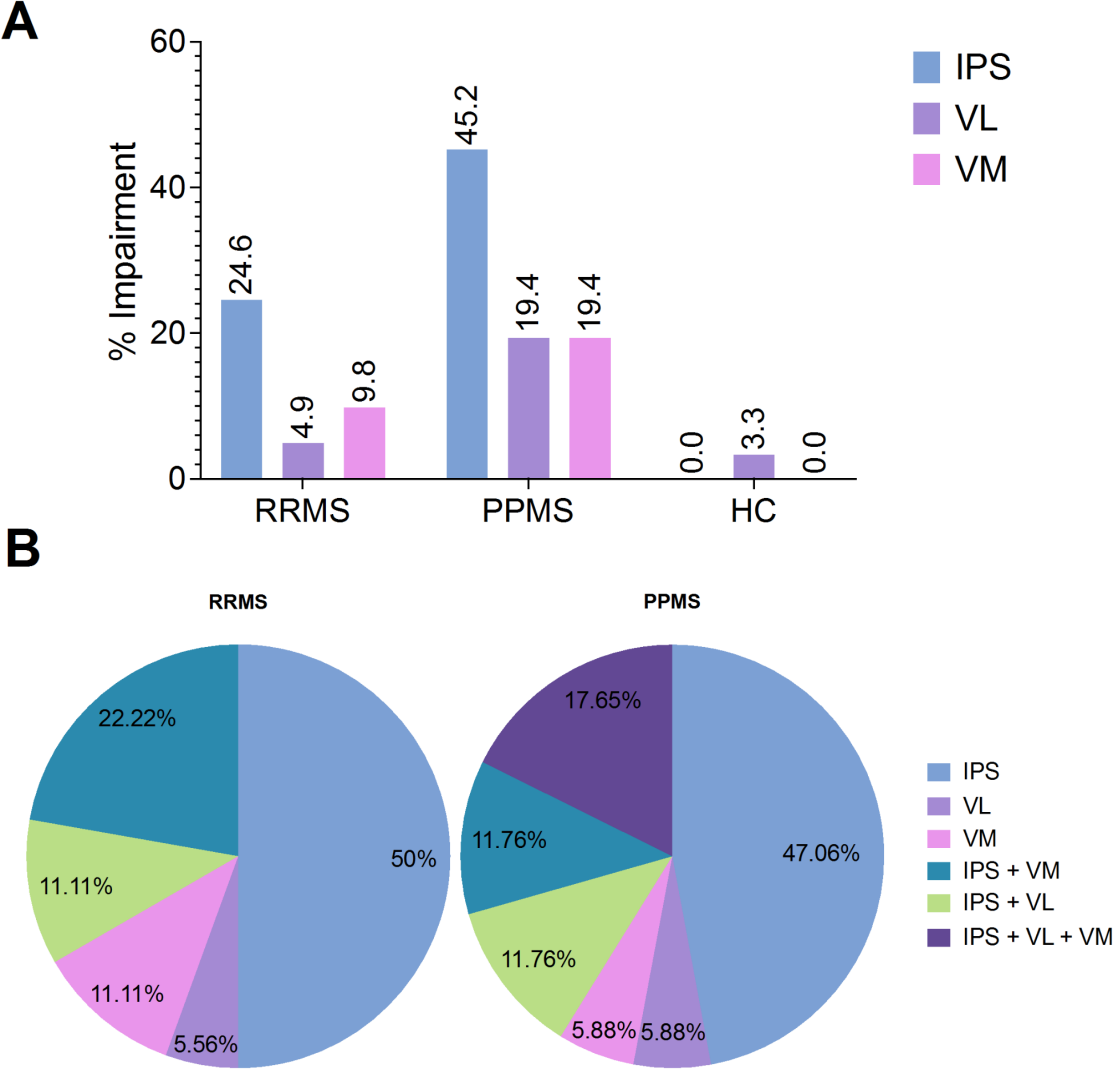
(**A**) Subgroup specific rates in information processing speed (IPS), verbal learning (VL) and verbal memory (VM). Impairment is defined as a z-score of 1.645 or more standard deviations below the mean of test-specific normative data. (**B**) Overview of the nature of cognitive dysfunction for all cognitively impaired pwRRMS (*n* = 18) and pwPPMS (*n* = 17).

Figure 1A shows the relative number of participants of each group with CI in IPS, VL, and VM. The nature of cognitive dysfunction for all cognitively impaired pwRRMS (*n* = 18) and pwPPMS (*n* = 17) is presented in Fig. 1B.

In pwRRMS, IPS was the most frequently impaired domain, whereas VL was the least frequently affected domain. Consistent with this, isolated IPS deficits were observed in 50% of pwRRMS with CI, whereas isolated VL or VM deficits rarely occurred. Any combination of two-domain impairment included IPS impairment and none of the pwRRMS had impairments in all three domains.

Similar to pwRRMS, IPS was the most frequently affected domain in pwPPMS. Deficits in VL and VM were equally common. Isolated IPS deficits were observed in 47% of pwPPMS with CI, while 5.9% presented isolated impairments in VL or VM. In addition, 17.7% had simultaneous deficits in IPS, VL, and VM. Again, any combination of two-domain impairment included IPS impairment.

Comparing impairment rates between pwRRMS and pwPPMS showed significantly higher rates in pwPPMS for IPS (one-sided *p* = .03, Φ = 0.21) and VL (one-sided *p* = .04, Φ = 0.23), but not for VM (one-sided *p* = .17, Φ = 0.13). In the HC group, no impairment was observed for IPS and VL. Only a minority (3.3%; *n* = 2) showed impairment in VM.

### Influence of IPS on VL and VM

Our aim was to investigate the influence of IPS on learning and memory performance as a possible explanation for differential impairment on measures of VL and VM. Table 3 shows the final regression weights and standard errors (SE) of our models predicting VL performance and VM in pwRMMS. We found that both VL performance and VM were significantly predicted by IPS in this group (β_VL_ = 0.47, *p <* .001; β_VM_ = 0.43, *p <* .001). Analyses revealed that the IPS regression weights were not significantly different between the two models (*Z* = 1.08, *p* = .28). In the PPMS group, IPS was also significantly associated with both VL and VM performance (β_VL_ = 0.73, *p <* .001; β_VM_ = 0.76, *p < .*001) (Table 4). Similarly, no significant differences were found in the beta weights of VL and VM (*Z* = 0.30, *p* = .94). When comparing the regression weights of VL and VM between the two MS groups, no significant differences were found (*Z*_*VL*_ = 0.62, *p* = .53; *Z*_*VM*_ = -1.51, *p* = .13) (Table S5). Table 5 shows that in the HC group, IPS did not predict VM (β = 0.05, *p* = .45) but did predict VL performance (β = 0.21, *p* = .02). Despite the relatively large difference between the standardised coefficients, their statistical comparison did not reveal a significant difference (*Z* = 1.41, *p* = .16).

**Table 3:** Summary of multiple regression analyses predicting verbal learning (VL) and verbal memory (VM) performance in pwRRMS (*n* = 59), and comparison of individual predictors and model fit across VL and VM.

Predictor	VL					VM					Comparison VL vs. VM		
	b	SE_b_	β	T	*p*	b	SE_b_	β	T	*p*	SE_b−diff_	Z	*p*
(Constant)	− 0.992	1.042		-0.952	0.345	0.528	0.792		0.666	0.508	1.31	-1.16	0.246
IPS	0.581	0.164	0.419	30.553	**< 0.001**	0.340	0.143	0.277	2.380	**0.021**	0.218	1.08	0.280
Sex	0.302	0.335	0.105	0.900	0.372	0.298	0.308	0.117	0.969	0.337	0.455	0.009	0.992
Age	0.026	0.018	0.173	1.477	0.146	− 0.021	0.018	− 0.157	-1.186	0.241	0.025	1.846	0.064
Depression	− 0.220	0.210	− 0.112	-1.044	0.301	0.004	0.255	0.003	0.017	0.986	0.330	−0 0.678	0.500
Fatigue	0.413	0.387	0.135	1.066	0.291	− 0.091	0.312	− 0.034	− 0.292	0.771	0.497	1.014	0.311
Education	0.435	0.305	0.206	1.426	0.160	0.231	0.293	0.124	0.789	0.433	0.423	0.482	0.630
*R²*	0.293					**0.**141							
*Adjusted R²*	0.211					0.042							
*F*	3.59					1.42							
*p*	**0.005**					0.23							

*p*-values < 0.05 are in boldface. *p*-values of model comparison based on two-tailed tests if not indicated otherwise. All SE are robust SE based on HC1-method. All T- and *p*-values derived from robust SE. IPS measured as SDMT z-score. Depression coded as 0 = normal, 1 = marginal, 2 = clinically meaningful; Fatigue coded as 0 = normal, 1 = clinically meaningful; Education coded as highest school-leaving qualification with 0 = none or lowest secondary school diploma (Hauptschule; ≤ 9 years, 1 = intermediate secondary school diploma (Mittlere Reife, 10–11 years), 2 = highest secondary school diploma (Fachabitur/Abitur, 12/13 years, regional differences); Age in years; Sex coded as 0 = male, 1 = female.

**Table 4:** Summary of multiple regression analyses predicting verbal learning (VL) and verbal memory (VM) performance in pwPPMS (*n* = 24), and comparison of individual predictors and model fit across VL and VM.

Predictor	VL					VM					Comparison VL vs. VM		
	b	SE_b_	β	T	*p*	b	SE_b_	β	T	*p*	SE_b−diff_	Z	*p*
(Constant)	1.794	1.397		1.284	0.216	-1.980	1.302		-1.521	0.147	1.910	1.976	**0.048**
IPS	0.732	0.179	0.682	4.087	**< 0.001**	0.757	0.237	0.664	3.197	**0.005**	0.297	−0 0.08	0.936
Sex	1.064	0.408	0.393	2.610	**0.018**	0.570	0.499	0.198	1.143	0.269	0.645	0.766	0.444
Age	− 0.031	0.019	− 0.279	-1.698	0.108	0.016	0.018	0.136	0.900	0.381	0.026	-1.796	0.072
Depression	− 0.394	0.323	− 0.168	-1.221	0.239	0.487	0.441	0.196	1.105	0.285	0.547	-1.612	0.107
Fatigue	− 0.266	0.432	− 0.098	− 0.615	0.547	0.134	0.531	0.047	0.253	0.803	0.685	−0 0.584	0.560
Education	0.432	0.252	0.220	1.712	0.105	0.681	0.277	0.326	2.458	**0.025**	0.374	− 0.665	0.506
*R²*	0.611					0.510							
*Adjusted R²*	0.474					0.337							
*F*	4.459					2.945							
*p*	**0.007**					**0.037**							

*p-*values < 0.05 are in boldface.* p*-values of model comparison based on two-tailed tests if not indicated otherwise. All SE are robust SE based on HC1-method. All T- and *p*-values derived from robust SE. IPS measured as SDMT z-score. Depression coded as 0=normal, 1= marginal, 2=clinically meaningful; Fatigue coded as 0= normal, 1=clinically meaningful; Education coded as highest school-leaving qualification with 0= none or lowest secondary school diploma (Hauptschule; ≤ 9 years, 1= intermediate secondary school diploma (Mittlere Reife, 10-11 years), 2= highest secondary school diploma (Fachabitur/Abitur, 12/13 years, regional differences); Age in years; Sex coded as 0=male, 1=female.

**Table 5:** Summary of multiple regression analyses predicting verbal learning (VL) and memory (VM) performance in HC and comparison of individual predictors and model fit across VL and VM (*n* = 61 for VL model and *n* = 60 for VM model^a^).

Predictor	VL					VM					Comparison VL vs. VM		
	b	SE_b_	β	T	*p*	b	SE_b_	β	T	*p*	SE_b−diff_	Z	*p*
(Constant)	0.910	0.568		1.602	0.115	0.880	0.496		1.781	0.081	0.753	0.040	0.968
IPS	0.213	0.088	0.264	2.420	**0.019**	0.054	0.070	0.072	0.779	0.449	0.112	1.414	0.157
Sex	0.304	0.199	0.183	1.524	0.133	0.030	0.211	0.019	0.143	0.887	0.290	0.945	0.345
Age	−0 0.004	0.008	−0 0.069	−0 0.472	0.640	−0 0.017	0.007	−0 0.315	-2.500	**0.016**	0.011	1.223	0.221
Depression	− 0.341	0.287	− 0.076	-1.19	0.239	-2.800	0.173	− 0.474	-16.213	**<0.001**	0.335	7.338	**<0.001**
Fatigue	− 0.642	0.228	− 0.143	-2.821	**0.** **007**	-1.245	0.242	− 0.211	-5.141	**<0.001**	0.332	1.814	0.070
Education	0.039	0.193	0.023	0.193	0.843	− 0.051	0.150	− 0.031	−0 0.338	0.737	0.244	0.368	0.713
*R²*	0.163					0.40							
*Adjusted R²*	0.070					0.332							
*F*	1.748					5.881							
*p*	0.128					**<0.001**							

*p*-values < .05 are in boldface.* p*-values of model comparison based on two-tailed tests if not indicated otherwise. All SE are robust SE based on HC1-method. All T- and *p*-values derived from robust SE. IPS coded as SDMT z-score. Depression coded as 0=normal, 1= marginal, 2=clinically meaningful; Fatigue coded as 0= normal, 1=clinically meaningful; Education coded as highest school-leaving qualification with 0= none or lowest secondary school diploma (Hauptschule; ≤ 9 years, 1= intermediate secondary school diploma (Mittlere Reife, 10-11 years), 2= highest secondary school diploma (Fachabitur/Abitur, 12/13 years, regional differences); Age in years; Sex coded as 0=male, 1=female. ^a^one participant was identified as an outlier and excluded from analyses in VM model computation.

## Discussion

The aim of this bicentric study was to investigate the influence of IPS on learning and memory performance as a possible explanation for differential impairment on measures of VL and VM. Our analyses revealed significant CI in both pwRRMS and pwPPMS when compared with age-matched HC. Consistent with previous research, our results suggest that IPS impairments represent the core cognitive deficit in both groups of pwMS^3,5,6^. In contrast, VL and VM performance were less commonly impaired. Although HC outperformed pwRRMS in VL, this was likely due to the above-average performance of our HC group, as indicated by a median z-score of + 1.0. The median RRMS z-score of + 0.5 indicated unimpaired VL, with only three persons exceeding the CI cut-off in this domain. This is consistent with previous research suggesting preserved VL performance in pwRRMS^28^. For VM, no significant difference was observed in pwRRMS. Conversely, HC significantly outperformed pwPPMS in both VL and VM, and median z-scores were negative for all domains, although not falling below the CI cut-off. This indicates that, despite individual variability, our cohort was rather mildly affected.

When comparing both MS groups, pwPPMS were outperformed by pwRRMS in all domains, with the most pronounced discrepancies observed in VL, as supported by the literature^7^. Regarding the prevalence of CI, our hypothesis of higher impairment rates in pwPPMS was only supported for IPS and VL, but not for VM. It is important to note that our primary research question focused on the influence of IPS on VL and VM. Therefore, we matched the HC to the RRMS and PPMS groups separately, rather than matching the RRMS and PPMS cohorts to each other. Thus, demographic and clinical differences, particularly the older age of the PPMS cohort, must be considered when comparing CI between the two groups, as (older) age is associated with non-relapse-related progression and cognitive decline^40^. However, as CI was defined based on age-corrected normative data, it is unlikely that the difference in impairment rates and average normative test performance was solely due to the age difference.

The observed differences in VL and VM performance between pwRRMS and pwPPMS are likely influenced by additional factors. First, a higher proportion of pwRRMS were receiving disease-modifying therapies, which may help prevent cognitive deterioration. Second, although MS is increasingly regarded as a continuum rather than a disease of discrete subtypes, pathophysiological processes vary over time^40^, potentially influencing cognitive outcomes. For instance, neurodegeneration has been shown to be more prominent in the progressive phase of the disease^41^, which may explain the poorer cognitive performance in pwPPMS.

Importantly, disease duration and depression levels did not differ significantly between the groups, suggesting these factors did not contribute to the differences in cognitive performance. While fatigue might have impacted neuropsychological test results^42^, it is notable that more pwRRMS reported clinically meaningful fatigue levels compared to pwPPMS, despite the latter group exhibiting worse cognitive performance across all tests.

In addition to these quantitative differences in CI, a threefold impairment of IPS, VL, and VM occurred only in the PPMS group (*n* = 3). This may indicate a phenotype specific pattern of CI, possibly due to greater cortical and subcortical damage in the PPMS group, as indicated by significantly higher EDSS scores and longer disease duration. However, given the lack of imaging data in our study, this remains speculative. Nevertheless, it is consistent with recent studies of cognitive phenotypes in MS, which have shown that severe multi-domain impairment is more common in pwPPMS compared with pwRRMS^43,44^.

To assess the relationship between IPS, VL, and VM, we first analysed the pattern and frequency of impairments in these domains. It was found that in both MS populations only a minority of participants exhibited deficits exclusively in VL (*n* = 1 per group) and VM (*n* = 2 RRMS and *n* = 1 PPMS). At the group level, both groups of pwMS were largely unaffected with respect to VL. Furthermore, none of the participants showed concurrent deficits in VL and VM without also having impairments in IPS. Thus, the reduced IPS in MS may indeed influence verbal learning and memory performance, in line with the *Cognitive Developmental Cascade Theory*^9^ and the *Relative Consequence Model.*^5^ However, in our regression analyses we observed a significant influence of IPS on both VL and VM in the MS groups, supporting the notion that IPS affects both learning and memory performance. When comparing the influence of IPS on VL and VM between pwPPMS and pwRRMS, no significant differences emerged, suggesting a similar influence of IPS on VL and VM across different MS phenotypes. Taken together, this suggests that the specific relationship between these functions is more complex than suggested by the *Relative Consequence Model*^5^ and requires a more nuanced view of cognitive processes in pwMS. Furthermore, it highlights the essential role of IPS for CI in pwMS, as discussed in the literature^3,4,6–8^ and extents recent findings by Chiaravalloti et al.^19^ which were limited to progressive phenotypes and VL.

In line with this, the regression analyses of the HC group suggest that VM is relatively independent of IPS, indicating that the cognitive mechanisms underlying the performance in this domain are different from those in pwMS. Our results support those recently presented by Mansical et al.^45^. They reported no significant correlation between IPS and memory in HC but a significant positive correlation in pwMS. While the HC group in Mansincal et al.^45^. may have been underpowered (*n* = 24), this does not apply to the present data. Our HC sample size was much larger, yet no significant effect was found.

In terms of practical implications, the results suggest the RAVLT learning trials alone cannot be equated with a comprehensive assessment of verbal memory. In both groups of pwMS, and particularly in the RRMS group, most participants with VM deficits could not be identified using trials 1 to 5 alone. The additional analysis of the delayed recall (VM) revealed memory deficits in *n* = 6 (9.84%) pwRRMS and *n* = 3 pwPPMS (9.68%) which might otherwise have gone undetected, emphasizing the need for a detailed examination to sufficiently assess memory functions in MS.

Clinicians using brief cognitive test batteries such as BICAMS should be aware that VL does not equal VM and that scores obtained from trials 1 to 5 are confounded by IPS. Particularly considering cognitive relapses^46^, it is important to accurately assess cognitive changes to detect any disease activity. Furthermore, the clinical implications of deficits in VL compared to VM have different impacts on daily functioning. For example, an individual may be able to learn a shopping list at home (indicating intact VL) but may struggle to recall the items 30 min later in the supermarket, reflecting a VM deficit.

Therefore, we recommend the implementation of Δ trial 5–7 as an important proxy for VM in both research and clinical practice. Given the significant VM deficits in the PPMS group, we advocate for the inclusion of specific memory training in routine patient care to help pwPPMS better cope with the challenges of CI in everyday life.

As this study focused exclusively on verbal long-term memory, we recommend that future research investigates possible impairments in short-term memory using relational measures (e.g. Δ trial 5–6 of the RAVLT). Building up on the *Developmental Cascade Theory*^9^ and the fact that short-term memory represents a lower stage in the cognitive hierarchy than long-term memory, deficits in IPS may have an even stronger impact on this memory domain. Future research should focus on the relationship between deficits in IPS, short- and long-term memory as the latter may be a precursor to the former. Prospective studies should also attempt to operationalise IPS in a more nuanced way than the SDMT to disentangle the contributions of different processing speed pathways. The common practice of equating cognitive IPS with results on the SDMT is problematic as IPS is influenced by three separate factors: sensory, cognitive and motor speed^47^. According to Costa et al.^24^, the SDMT is only suitable for measuring the combination of the three levels. However, it cannot assess the contribution of the different components of the processing pathway. Therefore, our results do not elucidate which specific aspect of IPS is deficient in pwMS and which aspect is responsible for the strong influence on VL and VM. Finally, the SDMT does not exclusively measure IPS, but is also influenced by other factors such as working memory, paired-associate learning, and visual scanning^3^.

Another interesting approach is to investigate the association of IPS with visuo-spatial memory using a comparable relational measure, such as the scores on the Brief Visuospatial Memory Test - Revised (BVMT-R)^48^. Although visual and verbal memory are distinct domains, each with different implications for everyday life, focusing on visual material in memory assessment may enhance the comparability of results across different countries and cultures. Preliminary data suggest that IPS, measured by the SDMT, significantly predicts visuospatial learning and memory performance in a mixed group of pwMS^49^. Notably, this association was specific to pwMS and was not observed in HC, which is consistent with our findings and previous results on verbal memory^45^. In a group of persons with primary or secondary progressive MS, the SDMT significantly predicted impairment in both verbal and visuospatial learning. The correlations between IPS and visuospatial learning were stronger than those for verbal learning, which could not be accurately predicted based on the SDMT^19^.

Investigating executive functions could also be a valuable direction for future research. However, unlike VL and VM, executive functions encompass a range of cognitive processes, such as planning, impulse control, and working memory, each of which requires distinct assessments. As a result, evaluating executive functions demands a more comprehensive test battery, as no single task can capture all aspects.

## Conclusion

In conclusion, our study highlights that pwPPMS have more pronounced deficits in IPS and VL compared to pwRRMS. Furthermore, we have demonstrated the pervasive impact of IPS on VL and VM performance in both pwRRMS and pwPPMS. IPS appears to affect both learning and memory, highlighting the importance of including IPS-focused interventions in the management of MS-related cognitive dysfunction. Importantly, cognitive changes in MS appear to be more complex than expected based on impairment rates, as the association between VM and IPS was only observed in pwRRMS and pwPPMS, but not in HC.

Our findings suggest that the RAVLT learning trials of the BICAMS alone cannot replace a comprehensive assessment of VM. In both patient groups participants with VM deficits could not be identified using trials 1 to 5 alone. Therefore, we recommend the implementation of Δ trial 5–7 as the primary memory measure in both research and clinical practice. Future research should further evaluate the relationship between IPS and verbal as well as visuo-spatial learning and memory using relational measures.

## Electronic supplementary material

Below is the link to the electronic supplementary material.

Supplementary Material 1

## Data Availability

The datasets used and/or analysed during the current study available from the corresponding author on reasonable request.
