# Supplementary material for: The influence of information processing speed on memory processes in patients with relapsing-remitting and primary progressive multiple sclerosis
Source: Sci Rep. 2025 Apr 8;15:11978. doi: 10.1038/s41598-025-96181-6 (PMC11978813; doi:10.1038/s41598-025-96181-6)
Supplement: Supplementary file 1 — Supplementary Material 1 [file 41598_2025_96181_MOESM1_ESM.docx]

**Supplementary Material**

**Supplementary Methods**

Equations used to compare regression weights across models:

To calculate SE_b-diff_ : SE_b-diff_= (SE_b(VL)_² + SE_b(VM)_²)^1/2^

To calculate *Z*: *Z*= (b_VL_ – b_VM_) / [(SE_b(VL)_² + SE_b(VM)_²]^1/2^

**Supplementary Tables**

Table S1
*Summary of backward multiple regression models predicting verbal learning performance and verbal memory in pwPPMS.*

| Predictor |  | Verbal learning | | | | |  | Verbal memory | | | | |
| --- | --- | --- | --- | --- | --- | --- | --- | --- | --- | --- | --- | --- |
|  |  | *b* | *SE_b_* | *β* | *T* | *p* |  | *b* | *SE_b_* | *β* | *T* | *p* |
| (Constant) |  | 1.60 | 1.18 |  | 1.35 | .19 |  | 0.32 | 0.37 |  | 0.87 | .40 |
| IPS |  | 1.08 | 0.25 | .72 | 4.42 | **<.001** |  | 0.68 | 0.20 | .59 | 3.45 | **.002** |
| Education |  | 0.51 | 0.29 | .27 | 1.75 | .10 |  |  |  |  |  |  |
| Age |  | -0.03 | 0.02 | -.28 | -1.81 | .09 |  |  |  |  |  |  |
| Sex |  | 1.31 | 0.42 | .50 | 3.15 | **.006** |  |  |  |  |  |  |
| *R²* |  |  | .60 |  |  | **.002** |  |  | .35 |  |  | **.002** |
| *F* |  |  | 6.61 |  |  |  |  |  | 11.93 |  |  |  |

*Note*. *p*-values<.05 are in boldface. Verbal learning model based on *N*=30 after exclusion of one outlier, Verbal memory model based on *N*=31. IPS measured as SDMT z-score. Education coded as highest school-leaving qualification with 0= none or lowest secondary school diploma (Hauptschule; ≤ 9 years, 1= intermediate secondary school diploma (Mittlere Reife, 10-11 years), 2= highest secondary school diploma (Fachabitur/Abitur, 12/13 years, regional differences); Age in years; Sex coded as 0=male, 1=female.

Table S2
*Summary of backward multiple regression models predicting verbal learning performance and verbal memory in pwRRMS.*

| Predictor |  | Verbal learning | | | | |  | Verbal memory | | | | |
| --- | --- | --- | --- | --- | --- | --- | --- | --- | --- | --- | --- | --- |
|  |  | *b* | *SE_b_* | *β* | *T* | *p* |  | *b* | *SE_b_* | *β* | *T* | *p* |
| (Constant) |  | 0.23 | 0.46 |  | 0.50 | .62 |  | 0.21 | 0.22 |  | 0.99 | .33 |
| IPS |  | 0.53 | 0.16 | .38 | 3.24 | **.002** |  | 0.36 | 0.16 | .30 | 2.33 | **.023** |
| Education |  | 0.54 | 0.25 | .25 | 2.15 | **.036** |  |  |  |  |  |  |
| *R²* |  |  | .23 |  |  | **.001** |  |  | .09 |  |  | **.023** |
| *F* |  |  | 8.27 |  |  |  |  |  | 5.44 |  |  |  |

*Note*. *p*-values<.05 are in boldface. Both models based on *N*=61. IPS measured as SDMT z-score. Education coded as highest school-leaving qualification with 0= none or lowest secondary school diploma (Hauptschule; ≤ 9 years, 1= intermediate secondary school diploma (Mittlere Reife, 10-11 years), 2= highest secondary school diploma (Fachabitur/Abitur, 12/13 years, regional differences).

Table S3
*Summary of backward multiple regression models predicting verbal learning and verbal memory performance in HC*

| Predictor |  | Verbal learning | | | | |  | Verbal memory | | | | |
| --- | --- | --- | --- | --- | --- | --- | --- | --- | --- | --- | --- | --- |
|  |  | *b* | *SE_b_* | *β* | *T* | *p* |  | *b* | *SE_b_* | *β* | *T* | *p* |
| (Constant) |  | 0.77 | .17 |  | 4.61 | **<.001** |  | 0.81 | .22 |  | 3.64 | .**001** |
| IPS |  | 0.24 | .10 | .29 | 2.36 | **.02** |  |  |  |  |  |  |
| Sex |  | 0.35 | .21 | .21 | 1.70 | .09 |  |  |  |  |  |  |
| Age |  |  |  |  |  |  |  | -0.02 | .01 | -.31 | -2.92 | **.005** |
| Depression |  |  |  |  |  |  |  | -2.76 | .62 | -.47 | -4.42 | **<.001** |
| Fatigue |  |  |  |  |  |  |  | -1.32 | .62 | -.22 | -2.14 | **.04** |
| *R²* |  |  | .12 |  |  | **.03** |  |  | .39 |  |  | **<.001** |
| *F* |  |  | 3.78 |  |  |  |  |  | 12.13 |  |  |  |

*Note*. *p*-values<.05 are in boldface. VL model based on *N*=61, TA model based on *N*=60 after exclusion of one outlier. IPS measured as SDMT z-score. Sex coded as 0=male, 1=female. Age in years. Depression coded as 0=normal, 1= marginal, 2=clinically meaningful; Fatigue coded as
0= normal, 1=clinically meaningful.

Table S4

*Summary of backward multiple regression models predicting verbal learning and verbal memory performance in pwMS*

| Predictor |  | Verbal learning | | | | |  | Verbal memory | | | | |
| --- | --- | --- | --- | --- | --- | --- | --- | --- | --- | --- | --- | --- |
|  |  | *b* | *SE_b_* | *β* | *T* | *p* |  | *b* | *SE_b_* | *β* | *T* | *p* |
| (Constant) |  | -0.21 | .38 |  | -0.57 | .57 |  | 0.28 | .19 |  | 1.50 | .14 |
| IPS |  | 0.61 | .12 | .47 | 5.14 | **<.001** |  | 0.51 | .12 | .43 | 4.23 | **<.001** |
| Education |  | 0.56 | .19 | .26 | 2.87 | **.005** |  |  |  |  |  |  |
| Sex |  | 0.61 | .26 | .21 | 2.3 | **.024** |  |  |  |  |  |  |
| *R²* |  |  | .34 |  |  | **<.001** |  |  | .18 |  |  | **<.001** |
| *F* |  |  | 13.28 |  |  |  |  |  | 17.91 |  |  |  |

*Note*. *p*-values<.05 are in boldface. Both models based on *N*=92. IPS measured as SDMT z-score. Education coded as highest school-leaving qualification with 0= none or lowest secondary school diploma (Hauptschule; ≤ 9 years, 1= intermediate secondary school diploma (Mittlere Reife, 10-11 years), 2= highest secondary school diploma (Fachabitur/Abitur, 12/13 years, regional differences); Sex coded as 0=male, 1=female.

| Table S5 *Comparison of individual predictors for verbal learning (VL) and verbal memory (VM) in pwRRMS (n=59) versus pwPPMS (n= 24).* | | | | | | |
| --- | --- | --- | --- | --- | --- | --- |
|  | Comparison VL RRMS versus PPMS | | | Comparison VM RRMS versus PPMS | | |
| Predictor | *SE_b-diff_* | *Z* | *p* | *SE_b-diff_* | *Z* | *p* |
| (Constant) | 1.74 | 1.60 | .11 | 1.52 | 1.65 | .10 |
| IPS | .24 | .62 | .53 | .28 | -1.51 | .13 |
| Sex | .53 | 1.44 | .15 | .59 | -.46 | .64 |
| Age | .03 | -2.18 | **.03** | .03 | -1.45 | .15 |
| Depression | .39 | -.45 | .65 | .51 | -.95 | .34 |
| Fatigue | .58 | -1.17 | .24 | .62 | -.37 | .71 |
| Education | .40 | -.01 | .99 | .40 | -1.12 | .26 |
| *Note*. *p*-values < .05 are in boldface. *p*-values of model comparison based on two-tailed tests if not indicated otherwise. All SE are robust SE based on HC1-method. All *T-* and *p*-values derived from robust SE. IPS coded as SDMT z-score. Depression coded as 0=normal, 1= marginal, 2=clinically meaningful; Fatigue coded as 0= normal, 1=clinically meaningful; Education coded as highest school-leaving qualification with 0= none or lowest secondary school diploma (Hauptschule; ≤ 9 years, 1= intermediate secondary school diploma (Mittlere Reife, 10-11 years), 2= highest secondary school diploma (Fachabitur/Abitur, 12/13 years, regional differences); Age in years; Sex coded as 0=male, 1=female. | | | | | | |
